# Early and certain Ménière’s disease characterization of predictors of endolymphatic hydrops

**DOI:** 10.3389/fneur.2025.1566438

**Published:** 2025-04-28

**Authors:** Joan Lorente-Piera, Víctor Suárez-Vega, Melissa Blanco-Pareja, Gloria Liaño, Octavio Garaycochea, Pablo Dominguez, Raquel Manrique-Huarte, Nicolas Pérez-Fernández

**Affiliations:** ^1^Department of Otorhinolaryngology, Clínica Universidad de Navarra, Pamplona, Spain; ^2^Department of Radiology, Clínica Universidad de Navarra, Madrid, Spain; ^3^Department of Otorhinolaryngology, Clínica Universidad de Navarra, Madrid, Spain; ^4^Department of Radiology, Clínica Universidad de Navarra, Pamplona, Spain

**Keywords:** Ménière disease, endolymphatic hydrops, 3D-real-IR, perilymphatic enhancement, endolymphatic space

## Abstract

**Introduction:**

Ménière’s disease (MD) is characterized by fluctuating sensorineural hearing loss, tinnitus, aural fullness, and episodic vertigo. Although endolymphatic hydrops (EH) is recognized as a pathognomonic finding, the relationship between clinical presentation, disease duration, and EH severity remains controversial, especially in early-stage MD. This study aimed to determine the impact of disease onset, temporal variables, and clinical phenotypes on EH severity and audiovestibular performance in a cohort with very early MD.

**Methods:**

We evaluated 80 patients diagnosed with certain MD, with symptom duration of less than 3 years. All underwent 3T MRI with 3D-real-IR sequences for EH evaluation, graded using cochlear (HCAFF), vestibular (HVAFF), and volumetric vestibular ratio (RELAFF). Multiple regression and correlation analyses were used to examine relationships between EH and clinical time markers (TD1, TS1, TR1, TR2), phenotypes, onset patterns, and audiovestibular outcomes (PTA, cVEMP, oVEMP, vHIT). Statistical significance was set at *p* < 0.05.

**Results:**

All patients exhibited some degree of EH, with 92.5% showing both cochlear and vestibular involvement. No significant correlations were found between time variables and EH severity. However, TS1 and TD1 were significantly associated with PTA in the affected ear (*r* = 0.277, *p* = 0.017 and *r* = 0.318, *p* = 0.006, respectively). Multinomial regression revealed that vestibular and complete onset patterns predicted more severe vestibular hydrops (HVAFF II and III: *p* = 0.019 and *p* = 0.020, respectively). The delayed phenotype was significantly associated with higher RELAFF values (*p* = 0.032), worse PTA in the affected ear (*p* = 0.005), and abnormal cVEMP responses (*p* = 0.002). No associations were found between phenotype or onset form and hydrops in general.

**Discussion:**

In very early MD, clinical time variables do not predict EH severity but correlate with hearing loss severity. The pattern of disease onset significantly impacts vestibular hydrops, whereas age correlates with cochlear EH, likely reflecting age-related hearing vulnerability. Phenotypic differences influence audiovestibular outcomes but not EH presentation. These findings highlight the diagnostic and prognostic utility of early symptom characterization and the need for standardization in defining disease duration in MD research.

## 1 Introduction

Ménière’s disease (MD) is a disease of the membranous inner ear characterized by recurrent fluctuating sensorineural hearing loss (HL), tinnitus, and aural fullness that occur during the episodes of vertigo with very significant changes in hearing and equilibrium (1). As the disease progresses, it generates a functional deficit, auditory first and vestibular later, which becomes irreversible (2). Additionally, it induces a significant deterioration in the patient’s quality of life due to the repetition of vertigo attacks, which eventually largely subside, giving way to instability that shows very diverse degrees of functional impact on the patient (3). The burden of this disease in specialized consultations, emergency rooms, or general medicine is constant due to its chronic nature and unpredictable course, estimating that they represent 12.8% of general Otorhinolaryngology consultations, 2.9% of general consultations, and 0.8% of emergency consultations (4, 5).

It is considered a rare disease, and difficult to provide a single figure describing its presentation as that depends on the environment in which the analysis is conducted. Reported prevalence rates for MD range from 3.5 per 100,000 to 513 per 100,000. For example, in the Spanish region of Cantabria the incidence was of 3 patients/100,000 inhabitants/year and the prevalence of 75/100,000, with a male-to-female ratio of 0.38 (6). A recent study using health claims data for more than 60 million patients in the United States found prevalence of 190/100,000 (7). Regarding the age, it is not common in individuals under 18 years old and it shows an incidence peak at 35–55 years old; however, in recent records the frequency in individuals over 65 years old has increased.

MD is a genetically caused and rare condition. Approximately 10% of cases are considered *familial* and follow an autosomal dominant inheritance pattern, with incomplete penetrance and variable expressivity, which leads to ethnic differences in prevalence. In the remaining cases, *sporadic*, it is common to find other family members affected by various types of otological diseases and inner ear alterations. The complexity of the etiology is based on its strong association with other processes (migraine, autoimmunity, autoinflammation), some of which, rather than mere comorbidity factors, can be true triggers; additionally, some of these exhibit a bidirectional influence: migraine, autoimmune diseases, asthma, hypovitaminosis D, osteoporosis (8). In the familial form, the most common alteration occurs with genes that mark the ultrastructure of the inner ear: OTOG encoding the Otogelin protein (which anchors the tectorial and otolithic membranes, as well the cupula to the hair cells and supporting cells), alpha-tectorin, and MYO7A among others (9). In the sporadic form, anomalies have been detected in genes that are also altered in patients with sensorineural hearing loss, as well as others that encode proteins for directed axonal growth. These patients also show multiple epigenetic alterations involved in the regulation of the inflammatory and immune response, leaving them in a state of increased susceptibility to developing an aggressive inflammatory process and, ultimately, endolymphatic hydrops due to stress, which could also explain two characteristics of the disease: its progressive nature and fluctuations (10). So far, no specific markers have been found, however patients with MD have higher basal levels of proinflammatory cytokines including IL-1β, IL-1RA, IL-6 and TNF-*α* when compared to healthy controls (11). Recently DNA methylation studies (one of the main epigenetic modifications of the human genome) open a more interesting path to differentiate patients from normal subjects.

A very intriguing part of the disease is its onset. When considering the onset of symptoms, MD can be divided into two subgroups: the typical group in which the patient presents the fundamental symptomatic triad, including vestibular and hearing symptoms as well as a feeling of auditory pressure, simultaneously from disease onset. In the atypical group, which is more frequent, MD does not begin with all three symptoms simultaneously. The most common initial symptom, when disease begins monosymptomatically, is vertigo (13.7%); however, when disease starts with two simultaneous symptoms, the most common pair (19.8%) is hearing loss and tinnitus (cochlear symptoms). The time elapsed from the appearance of the first symptom of MD until completion of the symptomatic triad also varies considerably across studies, in the same work the authors concluded that when MD had a monosymptomatic onset, the median time to completion of the symptomatic triad was 3 years (25th percentile 1.3 years and 75th percentile 6.9 years). When disease onset was bisymptomatic, median time to completion of the symptomatic triad was 2 years (25th percentile 0.74 years and 75th percentile 4.9 years) (12).

Once the disease is stablished three behavioral or evolutionary models based on the number of vertigo episodes per year experienced during the first 10 years of the disease have been described: (1) the patients experience episodes of vertigo in consecutive years during the first 4 years of the disease, (2) episodes of vertigo are experienced in the first 4 years of the disease followed by a period with no episodes that lasted for at least 1 year, with a subsequent relapse or occurrence of episodes of vertigo in one or more consecutive years, (3) there are multiple relapses throughout the course of the disease. This last model is also associated with a worse hearing prognosis and great number of Tumarkin’s otolithic crisis (13).

In patients with MD, the common histopathological finding is endolymphatic hydrops (EH), which is the dilation or distension of the membranous labyrinth due to an increase in the volume of endolymph relative to perilymph. Pressure-induced stress can affect virtually every part of the membranous labyrinth; making clinical manifestations neither uniform nor predictable. However, histologic alterations in animal models appear to follow a structured pattern, with a cochleo-centric progression: EH begins in the cochlear apex and extends to maculae and semicircular canals (14).

In humans, the fact that hydrops is cochleo-centric is a controversial issue. In the cochlea, histological lesions of secondary damage to hydrops (rupture of labyrinthine membranes) are common and appear to follow a very distinct cochleo-centric course. However, this contradicts with the hypothetical mission of Hensen’s duct (ductus reuniens), which connects the saccule and cochlea and the utriculo-endolymphatic valve (of Bast), which may regulate the reflux of endolymph toward the utricle, and otoconial remnants from the saccule (15, 16). Both structures could facilitate certain areas of the inner ear remaining unharmed during the evolutionary hydrops process. The endolymphatic sac also shows structural anomalies (degenerative or hypoplastic sacs) that can reduce endolymph reabsorption either by an epithelium alteration or accumulation of substances of indeterminate origin or electrolytes resulting in an osmotic influx of water (17). From a pathophysiological point of view, it is difficult to confirm the direct relationship between EH and MD, which raises doubts as to whether it is a mere epiphenomenon, but with greater repercussions on auditory and vestibular functional exploration, or it is a necessary *sine qua non* element upon which others must influence (18).

The most important diagnostic milestone achievement since 1938, when Yamakawa and Cairns and Hallpike discovery of EH in temporal bone study, was the visualization of EH in living patients using clinical MR imaging in 2007 by Nakashima (19, 20). Currently, two specific MRI sequences have been consolidated for the study of EH, known as MRI hydrops: the 3D-FLAIR sequence (fluid-attenuated inversion recovery) and the 3D-real-IR sequence (real inversion recovery reconstruction). Regarding Gadolinium (Gd) contrast, there are also two routes of administration: intravenous and intratympanic. However, there is a temporal limitation because the optimal time to obtain the MRI study is 4 h after a single dose of intravenous Gd and 24 h after intratympanic instillation. Although the 3D-FLAIR sequence is the most widespread, several publications highlight the superiority of 3D-real-IR in evaluating EH, especially cochlear EH, as it allows distinguishing endolymph (black signal) from the surrounding bone (intermediate gray signal) in the image (21). The current *in vivo* visualization of EH using MRI has enabled not only the assessment of its presence and its specific location within the membranous labyrinth but also the degree of EH within the inner ear. In addition to identifying the presence and extent of EH, other radiological findings associated with the disease have also been described, which are detailed below.

Other radiological findings associated to an endolymphatic hydrops are the perilymphatic enhancement (PE) probably due to an impairment of the blood–labyrinth barrier, which can be visualized in MRI as an intense contrast effect (22) and the herniation of the membranous labyrinth (HML) into the semicircular canals. Currently PE as a radiological marker is used in combination with EH grading system in patients suspected of having MD, in order to increase the positive predictive value in the diagnosis of definite MD (23). Recently, imaging study results of EH have been incorporated into the new diagnostic guidelines for Ménière’s disease. Thus, the definitive clinical form, in addition to audiological and vestibular criteria, must include the finding of EH to define the *certain* form of the disease (24).

Although a progressive increase of EH volume has been described in MD, there are currently not many specific radiological markers for early-stage involvement. Among the markers identified to date are the presence of isolated idiopathic saccular hydrops and saccule-to-utricle ratio inversion (SURI). SURI has been described using both semi-quantitative methods and by calculating the ratio of the sizes of the saccule and utricle in axial slices of a reference image (a ratio between the saccule area and utricle area ≥1 is considered positive). While both findings have also been observed in patients with more advanced stages of the disease, they are according to the authors more frequent in early stages and are associated with less severe cochlear and/or vestibular hydrops (25, 26).

In this study, we aimed to determine the best predictor of EH and, specifically, whether the time from the onset of initial symptoms to the MRI evaluation or the type of initial symptomatology presentation is a better indicator. The focus is on the earliest years following a diagnosis of *certain MD* particularly when the interval between the first symptoms and the MRI for hydrops is less than 3 years.

## 2 Materials and methods

### 2.1 Study design

The Research Ethics Committee of the University of Navarra (project number 2021.199) approved this study. All patients included in the study were recruited from two tertiary care center (Clínica Universidad de Navarra in Pamplona, Spain, and Clínica Universidad de Navarra in Madrid, Spain) over a study period spanning from 2018 to 2024. In accordance with ethical standards, all participants provided explicit consent for the use of their data for research purposes, and written informed consent was obtained from each subject prior to inclusion in the study.

### 2.2 Patient selection

- Inclusion criteria: all patients in this study were diagnosed of certain Ménière disease (MDcert). All of them fulfilled the criteria A 1(1–3) and B (1–5) of the Japan Society for Equilibrium Research (24). They were classified in the following phenotypic variants: idiopathic, delayed, autoimmune or with migraine (27). Because of the interest in this study, to focus on initial disease, all patients had to be scanned on the MRI in a period of time not longer than 3 years since the very first symptom.
- Exclusion criteria. familial and bilateral MD.

### 2.3 Examination and complementary tests

All patients underwent a physical examination, including otoscopy and otoneurological examination using a videonystagmography (VNG) system (VideoFrenzel Interacoustics VF505m, Assens, Denmark). The audiovestibular tests included pure tone audiometry (PTA) (AC40, Interacoustics), vestibular evoked myogenic potentials (VEMP, Eclipse Interacoustics, Assens, Denmark), Video Head Impulse Test (vHIT, ICS Impulse GN Otometrics® Natus Medical, Denmark) and when considered, the caloric test were also performed (VisualEyes™, Interacoustics, Denmark).

- Audiological evaluation: findings were reported in terms of pure tone average (PTA) thresholds from 0.25 to 6 kHz, expressed in decibels hearing level (dB HL).
- Vestibular evaluation: vHIT was used to analyze the gain of the vestibulo-ocular reflex, considering values below those expected by age and canal (28) as abnormal evaluating then the presence of corrective saccades (29). For VEMP, both cervical (cVEMP) and ocular (oVEMP) tests were conducted. An abnormal vestibular function was defined as a VEMP response in both ears with an interaural asymmetry ratio (IAAR %) exceeding 50%. Asymmetry ratios were analyzed for air-conducted stimulation at 0.5 kHz and 1 kHz with the acoustic stimulus intensity set at 97 dB normalized (30).
- All MR studies were performed at 3 Tesla magnets, either a Magnetom Vida or a Magnetom Skyra (Siemens Healthineers, Erlangen, Germany), with 20-channel and 32-channel phased-array receiver coils, respectively. A single dose of intravenous paramagnetic contrast agent gadobutrol (0.1 mmoL/mL, Gadovist, Bayer AG, Zurich, Switzerland) was administrated at a dose of 0.1 mL per kg of body weight. Images were acquired 4 h after the administration of contrast.
  - The imaging protocol consisted of:
  - A heavily T2 weighted sequence also referred to as *cysternography sequence* [T2 3D Sampling Perfection with Application optimized Contrasts using different flip angle Evolution (SPACE)] with the following parameters: section thickness, 0.5 mm; TR, 1400 ms; TE, 152 ms; flip angle, 120°; bandwidth, 289 Hz/pixel; voxel size, 0.5 × 0.5 × 0.5; and scan time, 5 min.
  - 3D-REAL-IR (inversion recovery with real reconstruction): section thickness, 0.8 mm; TR, 16,000 ms; TE, 551 ms; TI: 2700 ms; flip angle, 140°; bandwidth, 434 Hz/ pixel; voxel size, 0.5 × 0.5 × 0.8; and scan time, 11 min.

The 3D-REAL-IR sequence was chosen in favor of the 3D-FLAIR (fluid-attenuated inversion recovery) sequence as it exhibits a higher signal-to-noise ratio than the latter. (21).

The degree of cochlear hydrops (HCAFF) was determined using a three-level scale ranging from 0 to 2 (normal, mild, and severe), in an axial section passing through the modiolus and encompassing the greatest portion of the cochlea (19, 31). A four-level scale was used for the assessment of vestibular hydrops (HVAFF), the optimal visualization plane being the one displaying the greatest anatomical extent of the vestibule (32, 33).

The volumetric measurement of the vestibular endolymphatic ratio (RELAFF) was obtained dividing the endolymphatic volume (calculated in the 3D-REAL-IR sequence) by the total vestibule volume (calculated in the T2 cisternography sequence) and multipliying by 100. The volumes were measured in cm^3^ and the RELAFF was expressed in percentages. We defined Vestibular ELR values greater than 60% as indicative of radiologically significant vestibular hydrops, while values below 30% were categorized as radiologically non-significant vestibular hydrops (34).

### 2.4 Clinical evaluation

#### 2.4.1 Disease initiation classification

The study classified disease initiation based on the patient’s clinical history. Patients were categorized according to whether the disease began with simultaneous auditory and vestibular symptoms or whether one set of symptoms preceded the other for a prolonged period.

- Complete form: defined as the simultaneous onset of auditory and vestibular symptoms.
- Incomplete form: defined as the sequential onset of symptoms, with one domain (auditory or vestibular) preceding the other. This category was further subclassified into:
  - Auditory-initiated form: if the initial symptoms included hearing loss, tinnitus, or aural pressure before the onset of vertigo.
  - Vestibular-initiated form: if vertigo was the first symptom to manifest before auditory symptoms developed.

#### 2.4.2 Time periods

Several temporal variables were established to assess the progression of the disease:

- TD1 (latency to first true vertigo spell): Time elapsed from the first reported symptom to the first episode of true vertigo. This variable is particularly relevant for patients with an incomplete auditory-initiated form.
- TS1 (latency to second vertigo spell): Time elapsed between the first symptom (or group of symptoms) and the second vertigo episode, which meets the diagnostic criteria outlined in previous guidelines (35). This was further subclassified into:
  - *IATS1*: Incomplete auditory form
  - *IVTS1:* Incomplete vestibular form
  - *CTS1*: Complete form.
- TR1(time from second vertigo episode to mri for hydrops evaluation - MRIh): Interval between the second vertigo spell and the MRI assessment for endolymphatic hydrops.
- TR2: (time from first symptom to MRIh): Overall disease duration from the initial symptom onset to the MRI assessment for hydrops.

### 2.5 Clinical evaluation and follow up

The clinical follow-up and audiovestibular outcomes were assessed both before and after the definitive diagnosis of MDdef; in this study all data is as close as possible to the time in which the MRI was done. The onset and symptom progression were evaluated based on the mode of presentation, whether the symptoms appeared as a combination or as isolated auditory or vestibular symptoms. It is important to define that the reported vertigo episodes considered in the model refer to the time period within the last 6 months.

### 2.6 Statistical analysis

Descriptive statistical methods, including arithmetic means, standard deviations, and ranges, were employed for each group. In the statistical analysis the data of the patients with the delayed phenotype was not used. To conduct a multivariate analysis that links time variables with hydrops in a cross-sectional study, a multiple regression model was proposed, where the time variables (such as TS1, TD1, TR1, and TR2) serve as predictors, and the hydrops measurements (such as HcAFF, HvAFF, and RELAFF) are the dependent variables. Since this is not a longitudinal follow-up of patients but rather an analysis of data collected at a specific point in time, the aim is to identify the relationships between the time variables and the degree of hydrops at a static point. The formula we used, along with the interpretation of its variables, is as follows:

$\begin{array}{l} EHi=\beta 0+\beta 1\times TD1+\beta 2\times TS1+\beta 3\times TR1\\ +\beta 4\times TR2i+\beta 5\times N^{{}^{\circ}}\;\mathrm{Crises}+\beta 6\times \mathrm{Sex}\\ +\beta 7\times \mathrm{Age}+\beta 8\times \mathrm{Symptom}+\epsilon i \end{array}$

- TS1: Number of days between the onset of the first symptom (or set of symptoms) and the second vertigo episode that meets the diagnostic criteria A of the referenced guidelines.
- TD1: Number of days from the first reported symptom to the first confirmed vertigo episode.
- TR1: Number of days between the second vertigo episode and the MRI evaluation for endolymphatic hydrops (MRIh).
- TR2: Number of days between the initial symptom onset and MRIh.
- *β*₁, β₂, β₃, β₄, β₅, β₆, β₇, β₈: Regression coefficients representing the estimated effect of each predictor variable on the outcome.
- N° of Crises: Total number of vertigo episodes occurring in the last 6 months.
- Age: Patient’s age at the time of evaluation.
- Initial Symptom: Classification of the first symptom experienced by the patient, categorized as either auditory or vestibular.
- Sex: Binary variable where 0 represents male and 1 represents female.
- εᵢ (Residual/Error Term): Represents unexplained variability in the model due to unobserved factors.

A multinomial logistic regression model was conducted to explore the relationship between multiple independent variables: temporal, demographic, and disease presentation form, along the different dependent variables also presented as hydrops levels, both cochlear and vestibular, following the previously described formula. Variance inflation factors (VIFs) were computed for all independent variables, and those exhibiting high collinearity were examined. Variables with excessive collinearity were either combined, transformed, or removed to optimize model performance. Even more, although this assumption is primarily relevant for continuous predictors in logistic regression, potential deviations were evaluated, and transformations (e.g., logarithmic scaling) were considered where necessary. To ensure and optimize the interpretation of the model, both *p*-values and coefficients for the different variables were included.

Initially, a Fisher’s exact test was applied to study the possible association between the phenotype and the form of disease onset. For the RELAFF variable, a simple correlation analysis was applied using Pearson and Spearman coefficients to assess the direct relationship between temporal variables (TR1, TR2, TS1, TD1) and the levels of cochlear and vestibular hydrops. Spearman correlations were used for HCAFF and HVAFF due to the ordinal nature of the variables, while Pearson correlations were used for RELAFF, a continuous variable. Additionally, the Kruskal-Wallis test was applied to compare differences in TS1 and TD1 based on initial symptom onset and phenotypic variants. The same statistical method was used to analyze the relationship between the degree of HCAFF and HVAFF with audiovestibular tests, respectively, and to assess the relationship between the form of disease onset and audiovestibular test results.

Finally, to investigate whether there was a direct correlation between the times to the relative volume of endolymph to the vestibule (RELAFF) and to the results of the audiovestibular tests, a Pearson correlation model was applied for parametric variables and a Spearman correlation for non-parametric variables. The normality of the data distribution was evaluated using the Shapiro–Wilk test. A *p*-value of <0.05 was considered indicative of statistical significance. Statistical analyses were performed using Rstudio version 4.3.3 (Boston Massachusetts, United States).

## 3 Results

We have included 80 patients (47 male and 33 female) with a mean age of 55 ± 13 years. The demographic characteristics of the patients included in the study are summarized in Table 1.

**Table 1:** Summary of demographic data and clinical characteristics of the patients included in the study.

Clinical data	
Patients (Men:Women)	37 (46.25%)/ 43 (53.75%) Patients
Age (Mean±SD)	56.06 ± 12.58 Years
Disease duration (Mean±SD)	2.06 ± 4.20 Years
Affected side (Right:Left)	36 (45%)/ 44 (55%) Ears
Phenotype (idiopathic; migraine; autoimmune; delayed)	58 (72.5%): 12 (15%): 6 (7.5%): 4 (5%)

### 3.1 Form of presentation, timing and variants

In 27 cases (33.8%) all the symptoms appeared simultaneously but in 39 cases (48.8%) and 14 patients (17.5%) the initiation was monosymptomatic: auditory and vestibular, respectively.

**Figure 1:**
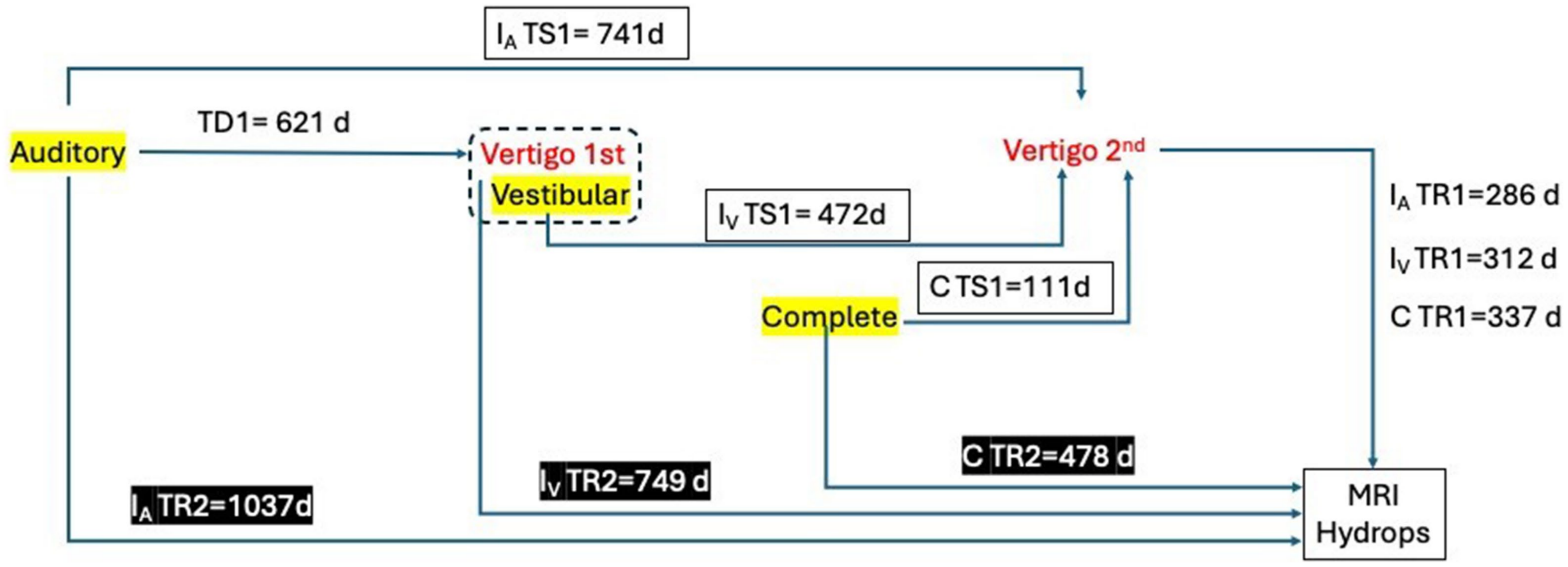
Summary of the different times in days included and studied in the sample. TD1: Time between the first symptom and the first true vertigo spell. TS1: Time between the first symptom (or collection of symptoms) and the second vertigo spell. IATS1: Incomplete auditory form. IVTS1: Incomplete vestibular form. CTS1: Complete form. TR1: Time between the second vertigo spell and MRI for hydrops evaluation (MRIh). TR2: Time between the first symptom and MRIh.

The periods of time for the non-delayed forms are shown in Table 2 and Figure 1, which summarize and represent the different times studied in our work. Only for this section, we excluded the 4 patients classified under the *delayed* variant phenotype from the analysis, as they exhibited inner ear damage with mostly hearing loss occurring years before the full MD symptom complex developed.

**Table 2:** Summary of the time periods in days based on each phenotype and disease initiation form.

Presentation	TD1	TS1	TR2	TR1
Initiation				
Incomplete Auditory (*N* = 35)	621 ± 62	741 ± 69	1,037 ± 120	286 ± 68
Incomplete Vestibular (*N* = 14)	9 ± 9	472 ± 126	749 ± 186	312 ± 56
Complete (*N* = 27)	0	111 ± 19	478 ± 89	337 ± 74
Phenotype				
Idiopathic (*N* = 58)	321 ± 63	519 ± 71	884 ± 80	373 ± 37
Migraine (*N* = 12)	218 ± 89	357 ± 81	588 ± 164	229 ± 106
Autoimmune (*N* = 6)	187 ± 62	353 ± 82	434 ± 103	79 ± 46
Summary (*n* = 76)	**226 ± 57**	**426 ± 81**	**694 ± 217**	**269 ± 68**

The different results are presented with the mean and standard deviation (Mean±SD).

Regarding the initiation of the disease, the time to the second vertigo spell (TS1) that marks the diagnosis as definite (given that all patients in our study got a hearing test showing fluctuation) was significantly different between groups (*p* < 0.001). However, when considering the clinical phenotype, no significant differences were found in any of the periods of time here analyzed.

Finally, as can be observed in Table 3, when analyzing the most frequent forms of presentation based on the phenotype, we found that for all groups, the main form of initiation was auditory, followed by complete, except for the delayed phenotype, in which none of the patients exhibited the combination of symptoms. Nonetheless, the result of Fisher’s exact test did not present statistically significant results (*p* > 0.05).

**Table 3:** Distribution of disease initiation form by phenotype in the study patients.

Debut onset	Idiopathic (58)	Migraine (12)	Autoimmune (6)	Delayed (4)	*p* value
Incomplete auditory	26 (44.82%)	6 (50%)	4 (66.67%)	3 (75%)	0.319
Incomplete vestibular	11 (18.97%)	1 (8.33%)	1 (16.67%)	1 (25%)	0.275
Complete	21 (36.21%)	5 (41.67%)	1 (16.67%)	–	0.999
Total	58	12	6	4	–

### 3.2 Radiological endolymphatic hydrops: findings by phenotype and timing

All patients had some degree of hydrops in the cochlea, in the vestibule or (more frequently as in 74/80) in both.

**Figure 2:**
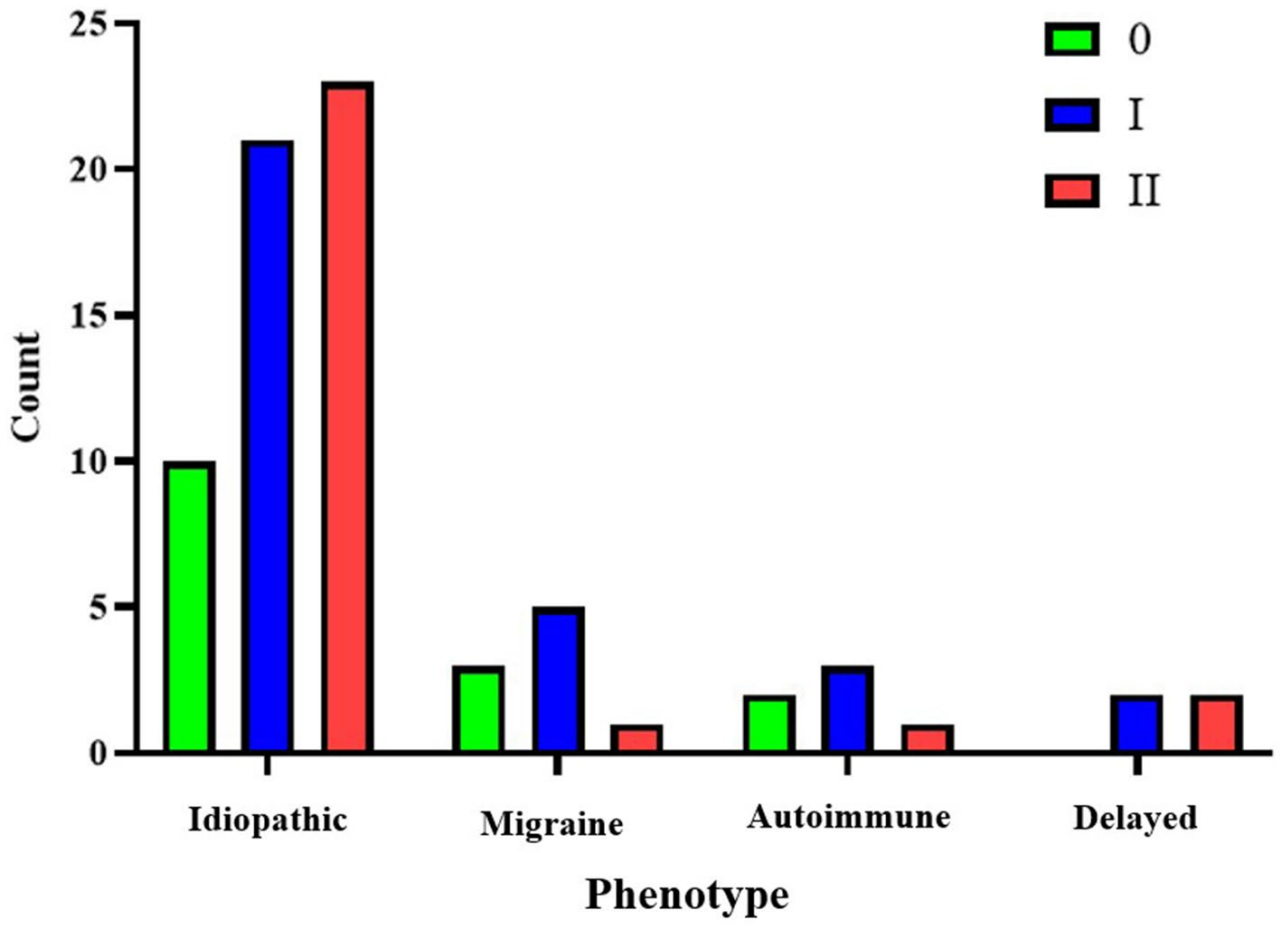
Representation of cochlear hydrops (HCAFF) grades for each phenotype.

**Figure 3:**
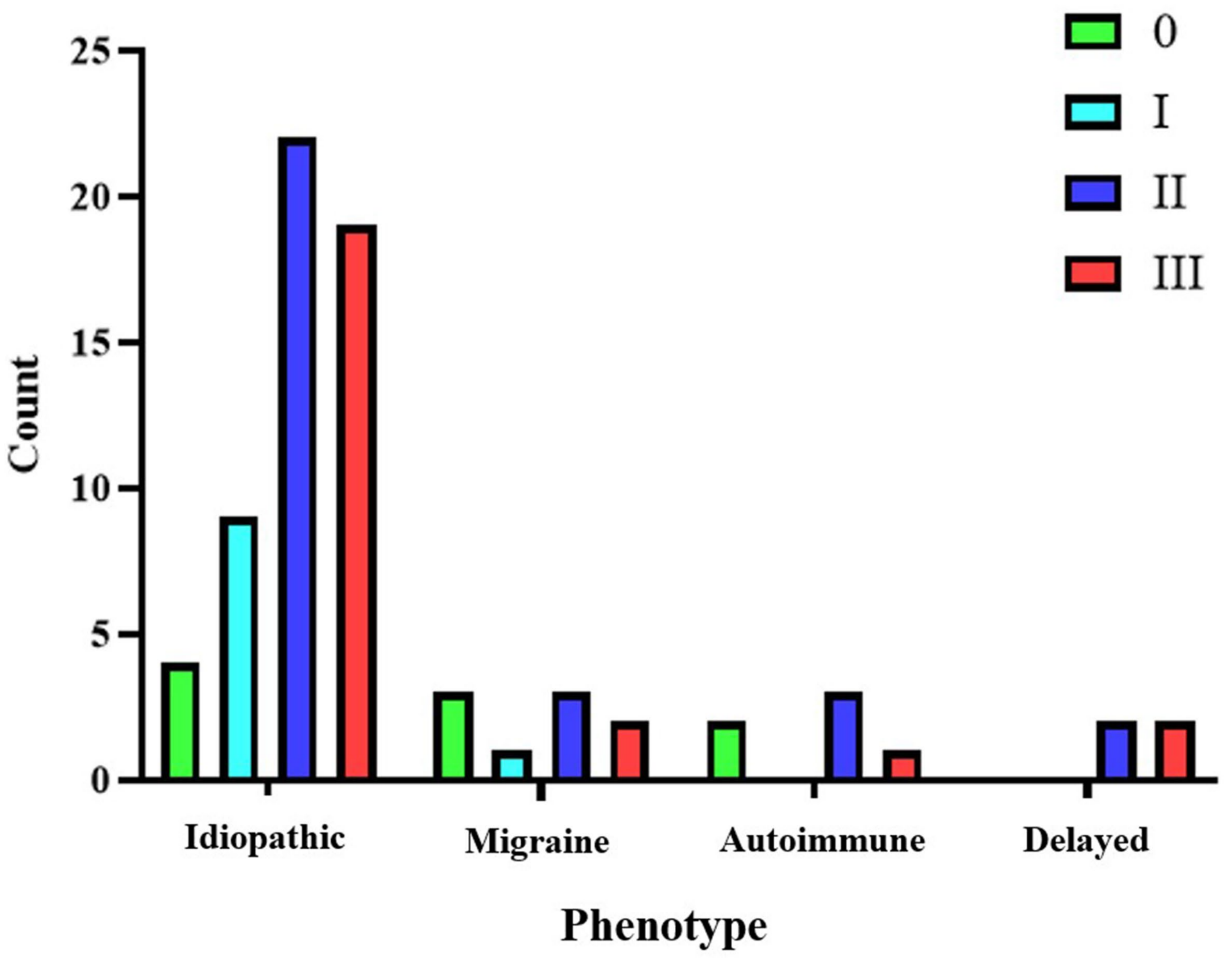
Representation of vestibular hydrops (HVAFF) grades for each phenotype.

For the idiopathic phenotype, which is the most prevalent in our cohort, the most common finding at the cochlea was a grade II or severe hydrops (42.59%). At the vestibule, the most frequent finding was grade II or moderate hydrops (40.74%), making it the typical presentation for this phenotype. In the second most common group, the migraine phenotype, the most frequent cochlear finding was grade I or mild hydrops (55.55%), while at the vestibule, grade II or moderate hydrops (33.33%) was the most typical presentation. A similar pattern was observed in the third most frequent group, the autoimmune phenotype, with 50% of cases showing grade I cochlear hydrops and the same proportion showing vestibular hydrops, where grade II or moderate hydrops was the most common finding. Finally, in the delayed phenotype group, the distribution was evenly split between grade I-II cochlear hydrops and grade II-III vestibular hydrops. Notably, none of the individuals in this subgroup presented with findings below a moderate grade for either cochlear hydrops (HCAFF) or vestibular hydrops (HVAFF). The breakdown of results and radiological classification according to the phenotype at diagnosis is represented in Figures 2, 3.

The Spearman correlation was applied, to study the individual direct relationship between the time variables (TS1, TD1, TR1, and TR2, along with their variants) and the radiological parameters defining hydrops severity in a qualitative manner using HCAFF and HVAF. No statistically significant results were observed. The correlation coefficients (*r* values) were significantly low, indicating a very weak or non-existent correlation.

### 3.3 Impact of timings and initiation on the severity of endolymphatic hydrops

**Figure 4:**
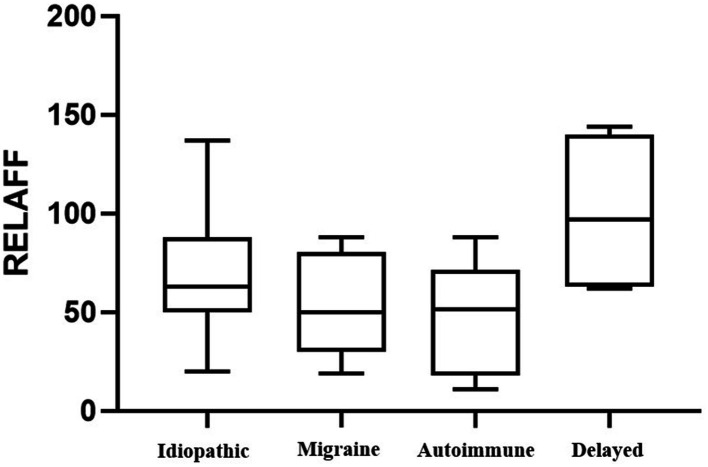
Representation of the volume of RELAFF for each phenotype in the study.

We then conducted a multinomial regression model. In our model the different periods of time do not show any significance to the severity of EH (Table 4). It is interesting to highlight that age was the most consistent variable providing statistically significant *p*-values in both the HCAFF and HVAFF models, specifically in the development of mild to moderate cochlear and vestibular hydrops. We have only found that the HVAFF grade shows statistically significant results in the model when the disease presents as incomplete vestibular or as complete, with statistically significant results for grades II and III. When analyzing the relative volume of endolymph to vestibule (RELAFF), as shown in Figure 4, by all clinical phenotypes, only the delayed form shows a significant difference (*p* = 0.032).

**Table 4:** Results of *p*-values from the multinomial analysis for HCAFF and HVAFF.

Variable	HCAFF (*p*-value/coefficient)		HVAFF (*p*-value/coefficient)		
	Grade I	Grade II	Grade I	Grade II	Grade III
TD1	0.649/0.635+	0.841/0.227+	0.307/0.299+	0.418/0.221+	0.622/0.135+
TS1	0.943/0.728+	0.769/0.256+	0.639/0.940+	0.360/0.174+	0.611/0.833+
TR1	0.890/0.121+	0.959/0.421+	0.699/0.536+	0.332/0.107+	0.921/0.119+
TR2	0.716/0.194+	0.619/0.214+	0.166/0.147+	0.605/0.530+	0.484/0.713+
Crisis (*N*)	0.347/0.003	0.126/0.028	0.623/0.002	0.296/0.004	0.590/0.001
Age (Y)	0.031*/0.025	0.382/0.010	0.004*/0.437	0.001*/0.125	0.390/0.013
Gender	<0.001*/0.877	0.002*/0.787	0/0.438	0.093/0.537	0.961/0.016
Inc. Auditory	0.511/0.588	0.799/0.233	0/0.711	0.466/0.286	0.622/0.193
Inc.Vestibular	0.869/0.175	0.522/0.695	0/3.424	0.019*/15.792	0.020*/13.962
Complete	0.563/0.752	0.341/0.336	0/0.179	0.012*/8.73	0.031*/2.08

* and bold values indicate statistically significant results (*p* < 0.05) and + indicates that the coefficient values have been multiplied by one thousand.

### 3.4 Audiovestibular test results and their impact on endolymphatic hydrops

**Figure 5:**
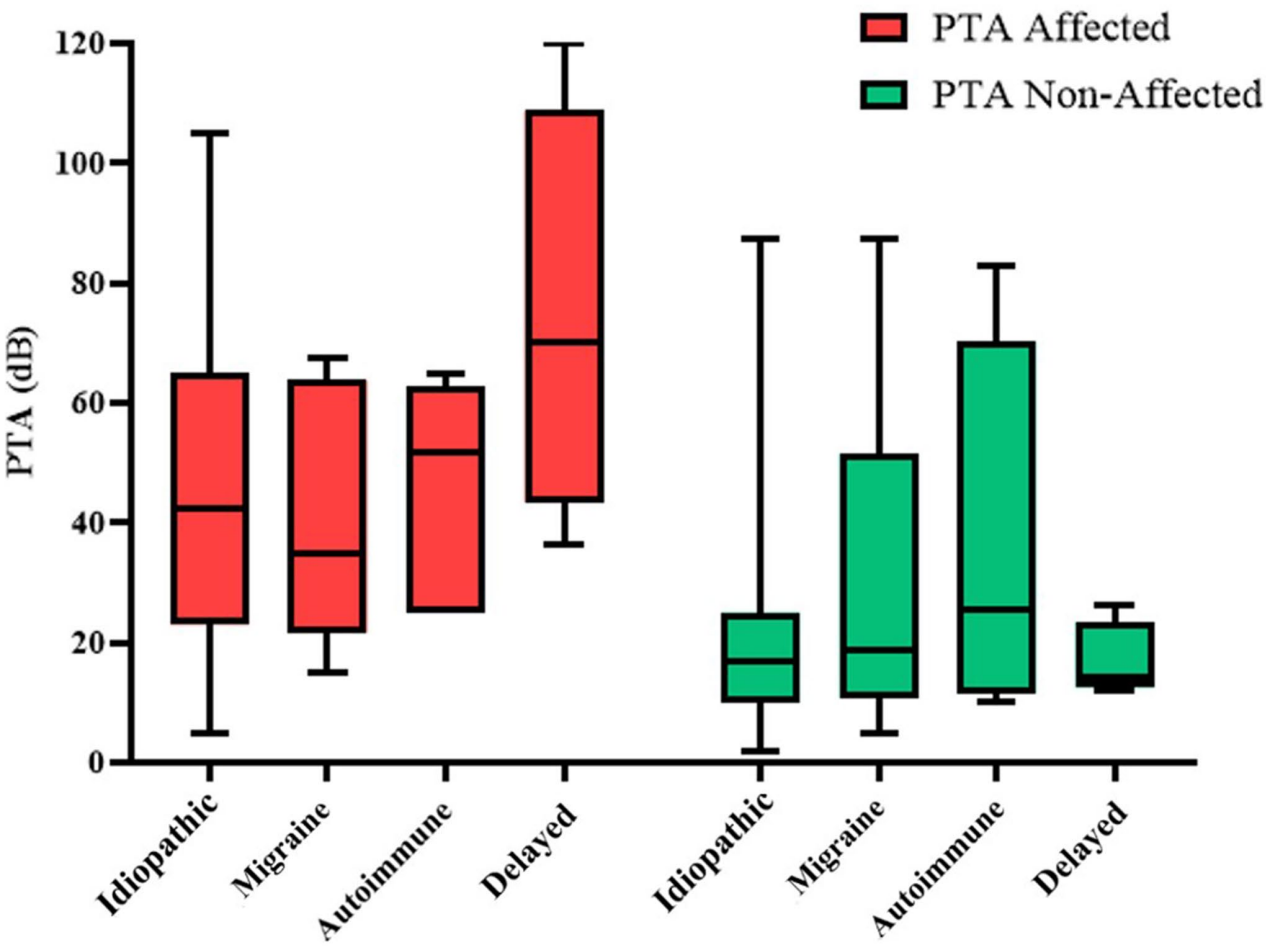
Representation of both PTAaff and PTANaff for each phenotype.

**Figure 6:**
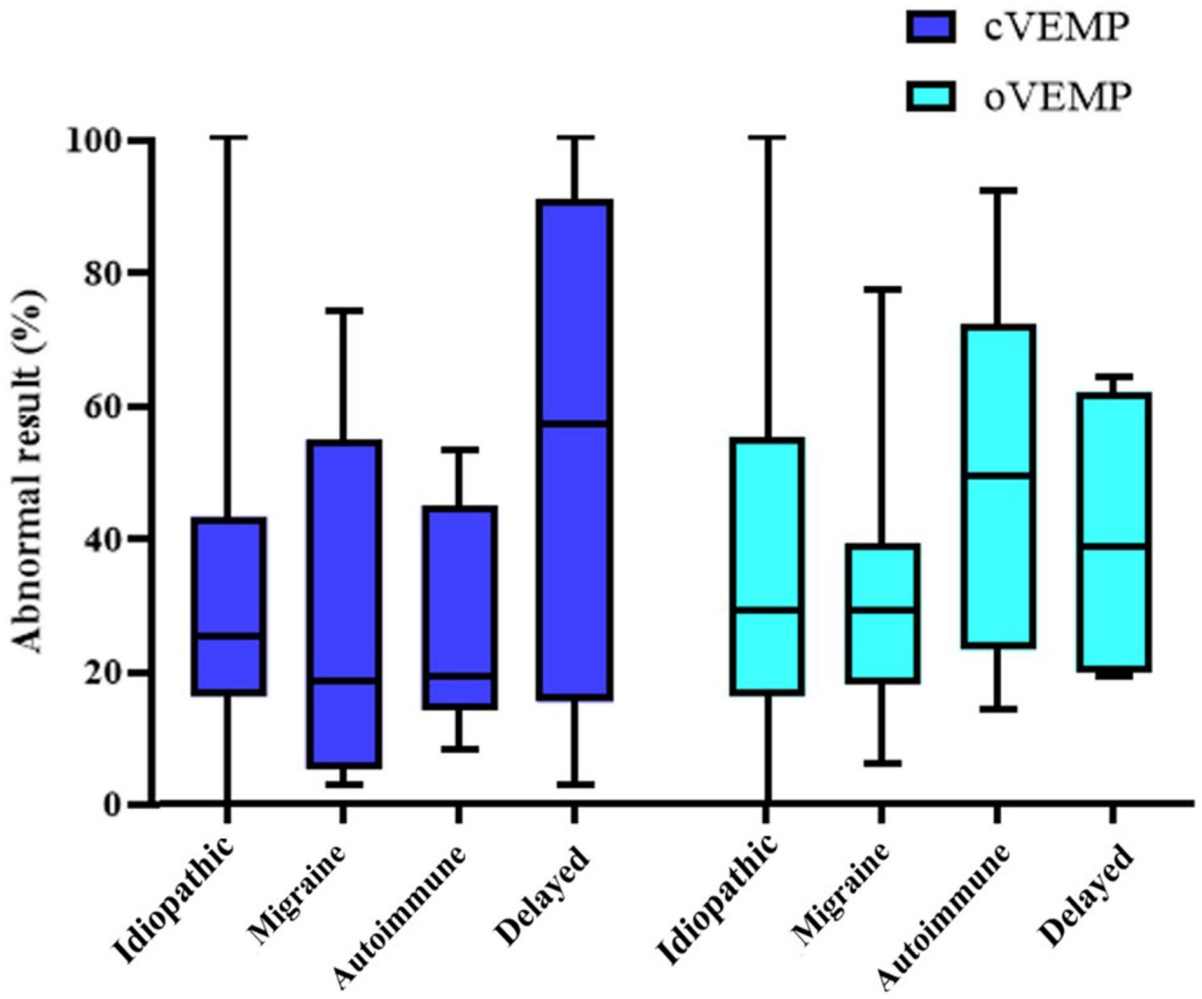
Representation of both cVEMP and oVEMP for each phenotype.

When analyzing the audiovestibular results explained in section 2.3, according to each phenotype, we obtained the findings summarized in Table 5 and Figures 5, 6.

**Table 5:** Mean results of the audiovestibular results for each phenotype.

Test	Idiopathic	Migraine	Autoimmune	Delayed	*p* value
PTAaff (dB)	43.6 ± 22.7	39.5 ± 19.3	46.8 ± 16.2	74 ± 30.1	0.005*
PTANaff (dB)	17.4 ± 12.8	13 ± 5.2	11.8 ± 5.3	16.7 ± 5.6	0.010*
oVEMP(%)	41.5	14.3	66.7	50	0.098
cVEMP(%)	50	50	16.6	75	0.002*
vHIT(%)	22.7	33	33.3	75	0.854

Aff, Affected ear; Naff, Non-affected ear; vHIT and VEMP are expressed as percentage (%) of abnormal results. The different results are presented with the mean and standard deviation (Mean±SD). * and bold values indicate statistically significant results (*p* < 0.05).

The phenotype with the worst auditory result in the affected ear is the delayed phenotype, with a PTA of 74 ± 30 dB; the intergroup difference is statistically significant (*p* = 0.005). On the non-affected side, the phenotype with the poorest audiometric results is the idiopathic phenotype, maintaining statistical significance (*p* = 0.010) but however in within the normal range. Regarding the results of vestibular tests, the delayed phenotype once again shows the worst outcomes across vHIT, oVEMP, and cVEMP tests. However, a statistically significant difference is only observed in the cVEMP results (*p* = 0.002). On the other hand, when we analyzed the relationship between the form of disease presentation and the results of audiovestibular tests, we did not find any statistically significant results in any of them.

In Table 6, the results of audiovestibular tests are presented based on the degree of endolymphatic hydrops, HCAFF or HVAFF. The results of the Kruskal-Wallis analysis showed statistical significance only for both HCAFF and HVAFF with the PTA of the affected ear (< 0.001).

**Table 6:** Summary of the relationship between the degree of HCAFF and HVAFF and the results of audiovestibular tests.

EH	Degree	PTAaff (dB)	PTANaff (dB)	cVEMP (%)	oVEMP (%)	vHIT (%)
HCAFF	0	27.02 ± 19.24	11.11 ± 6.09	39.50 ± 34.18	36.00 ± 33.19	20
	I	41.46 ± 23.88	15.30 ± 8.20	24.66 ± 17.72	38.86 ± 25.15	16
	II	58.72 ± 20.69	19.40 ± 15.12	40.20 ± 31.21	32.19 ± 22.37	14.81
	*p* value	<0.001***	0.116	0.160	0.487	0.623
HVAFF	0	27.23 ± 15.32	10.81 ± 7.67	28.86 ± 24.07	33.00 ± 23.43	11.11
	I	26.08 ± 10.99	11.58 ± 6.30	28.20 ± 27.77	42.30 ± 31.67	10
	II	42.00 ± 21.90	17.06 ± 10.97	29.42 ± 21.61	33.89 ± 24.26	26.67
	III	62.92 ± 23.27	18.33 ± 13.59	41.09 ± 33.42	35.73 ± 24.53	41.67
	*p* value	<0.001***	0.145	0.583	0.909	0.869

The different results are presented with the mean and standard deviation (Mean±SD). *** and bold values indicate statistically significant results (*p* < 0.001).

### 3.5 Impact of timings on audiovestibular test results

**Figure 7:**
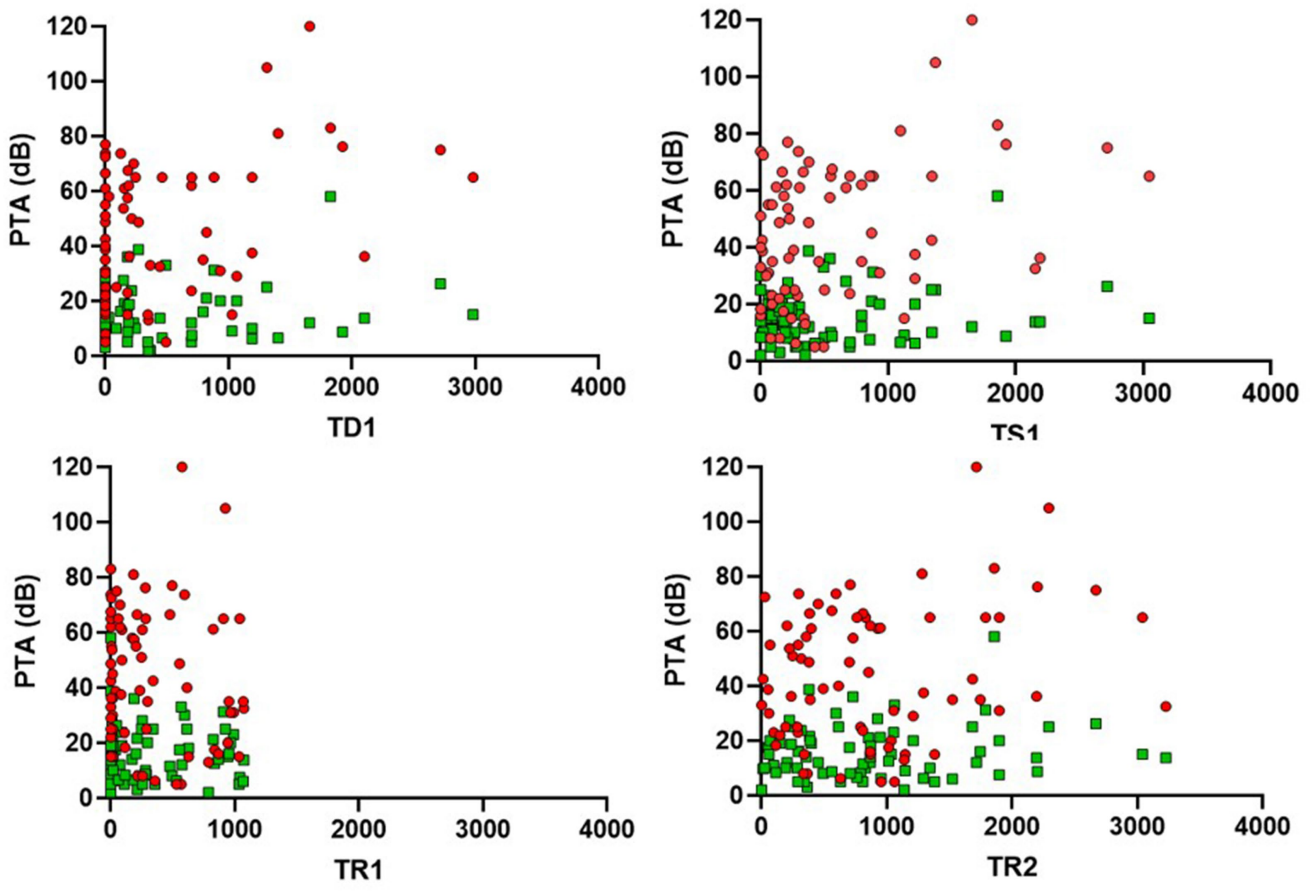
Scatter plots showing the relationship between PTA in the affected and non-affected ear and four clinical times (TD1, TS1, TR1, and TR2). The red dots refer to the PTA values of the affected ear, while the green dots correspond to those of the unaffected ear.

**Figure 8:**
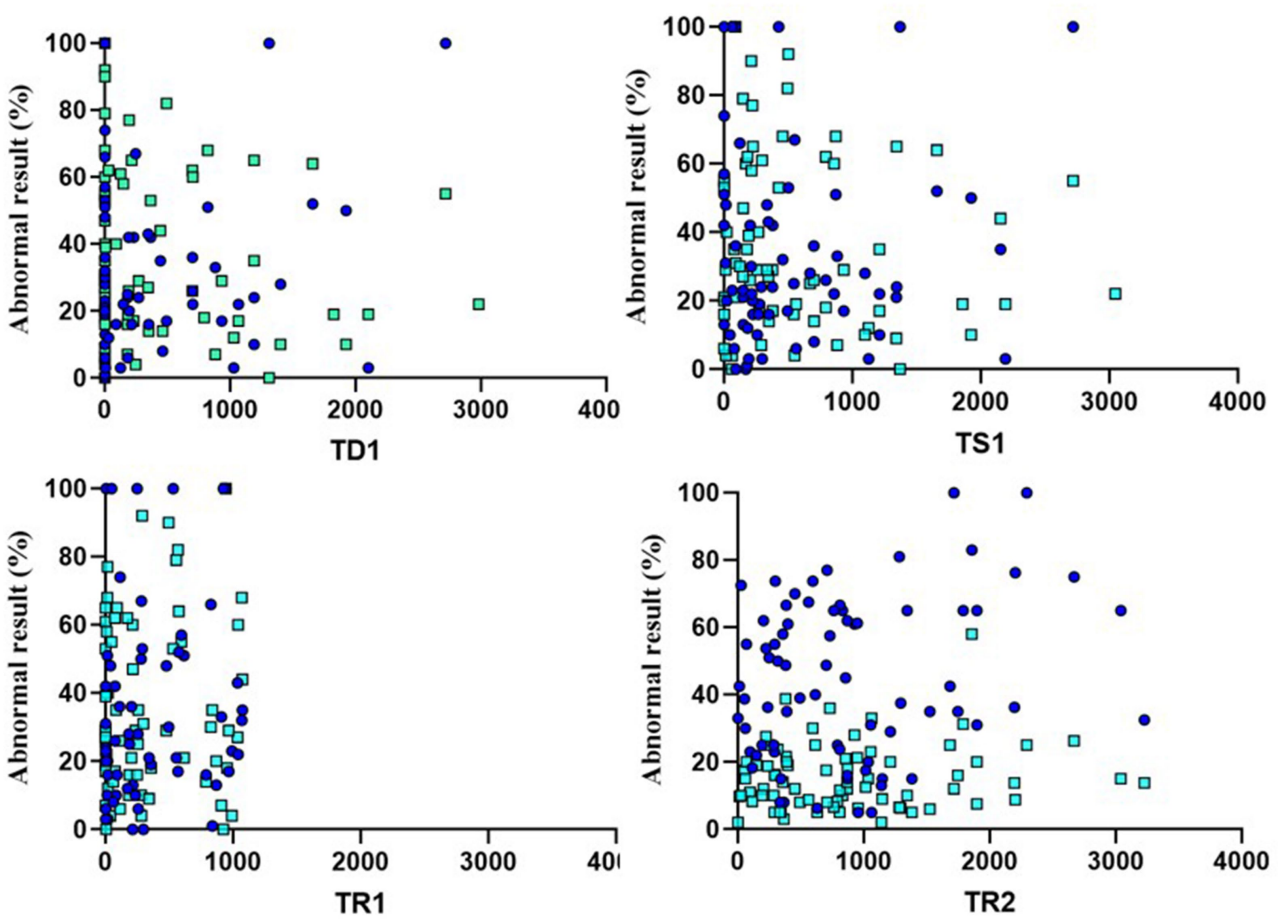
Scatter plots showing the relationship between VEMPS and four clinical times (TD1, TS1, TR1, and TR2). The dark blue dots refer to the cVEMP values of the affected ear, while the light blue dots correspond to those of the oVEMP.

In this section we analyzed how the different study times influenced the results of objective audiovestibular tests. We used simple correlation methods with tests that allowed us to observe how statistically strong the correlation between variables was and whether these differences reached statistical significance. Both results can be observed in Table 7 and Figures 7, 8.

**Table 7:** Summary of the correlation coefficients (*r* value) and *p*-values from the Spearman model to relate time points with the results of audiovestibular tests.

Test		TD1	TS1	TR1	TR2
PTAaff (dB)	*r* value	0.318	0.277	0.121	0.150
	*p* value	0.006*	0.017*	0.308	0.205
PTANaff (dB)	*r* value	0.040	0.002	0.056	0.090
	*p* value	0.732	0.988	0.636	0.446
oVEMP(%)	*r* value	0.091	0.075	0.052	0.090
	*p* value	0.456	0.542	0.668	0.446
cVEMP(%)	*r* value	0.018	0.009	0.216	0.150
	*p* value	0.884	0.940	0.080	0.204

* and bold values Indicate statistically significant results (*p* < 0.05).

In summary, the results indicate that only the time points TD1 and TS1 show statistically significant correlations with the values obtained in the tonal audiometry of the affected ear (PTAaff). In both cases, the correlation values are considered weak positive (0.318 for TD1 and 0.277 for TS1). None of the other clinical time periods showed relevant correlations neither with the other audiovestibular tests evaluated (PTANaff, oVEMP, cVEMP).

## 4 Discussion

In this study, we focused on patients with Ménière’s disease (MD) at very early stages to evaluate the degree of endolymphatic hydrops (EH). Previous research has shown that the extent of EH does not correlate with disease duration. This has been observed in studies combining definite and probable cases (36) or even possible cases (37), and that include patients with long disease duration (38). Conversely, some studies have reported a correlation between EH severity in the cochlea and vestibule with disease duration (32, 39). Notably, some studies included patients with short disease durations (approximately 2 years) alongside those with durations up to 20 years (40). A recent comprehensive study, which involved a cohort like ours, analyzed the duration of specific symptoms such as hearing loss, vertigo, aural fullness, and tinnitus; the authors found a non-linear correlation between the duration of vertigo and EH (41). Although this study also included patients with relatively short disease durations, its sample exhibited a wide distribution of disease duration.

Given the heterogeneity in inclusion criteria and disease duration across previous studies, as well as the lack of a standardized definition for disease duration, we first focused on patients with an unequivocal diagnosis of MD classified as *definite* under current guidelines. More specifically, we adhered to the “certain” category of the most comprehensive guidelines, which include not only general clinical characteristics but also evidence of auditory and vestibular dysfunction as well as imaging-confirmed hydrops. Additionally, we conducted a detailed analysis of the time intervals between symptom onset and diagnosis. This required the inclusion of patients with a short disease duration, as accurately recalling specific events (even those deemed significant) can be challenging for many individuals. To address this issue, we excluded patients unable to reliably provide a precise retrospective symptom timeline or for whom external validation was not possible (42). Consequently, we set a maximum period of symptoms of 3 years for inclusion in this study, except for patients with the delayed-onset variant of MD. This approach allowed us to minimize recall bias and ensure a more accurate assessment of the relationship between disease progression and the degree of EH. Considering the 3-year period for early diagnosis from the onset of the first symptom, the presence of signs of endolymphatic hydrops contributes to the diagnostic confirmation in the initial stages of the disease. Given that most patients may appear with an incomplete form, MRI plays a crucial role in the diagnostic process and suggest its implementation as a diagnostic measure for early stages when MD is part of the differential diagnosis.

Our study is closer to those who do not find any relation between disease duration and severity of EH. Neither the time form the first symptom to MRI (TR2) nor to the second vertigo crisis (TS1) showed any significant difference when assessing the severity of EH both in the cochlea and vestibule. TR2 was easily accessible from all patients and was however found to be well related to the type of initiation of the disease (complete or incomplete). TR1 is what should probably be better considered as disease duration because extends from the date of the vertigo crisis that marks the event defining the disease, to MRI. It indicates the accessibility of specialized consults and of MRI.

To be more precise a multinomial regression model was conducted to simultaneously evaluate the relationship between multiple independent variables (such as the different time periods TS1, TD1, TR1, and TR2, along with other variables like the number of crisis, sex, age, and initial symptom) and the dependent variables (HCAFF and HVAFF). This multivariable approach is essential for considering how multiple factors interact and contribute to the development of hydrops. Additionally, we adjusted for the effects of other variables, providing a comprehensive and refined analysis of the phenomenon. As seen in section 3.3 and in concordance to previous finding, none of the period of times correlates to severity of EH.

The pathophysiological explanation for these findings remains unclear, particularly when considering phenotypic variants. It can be hypothesized that initial disruption in the homeostasis of the inner ear is necessary for hydropic changes to be detected via resonance imaging. However, the primary mechanism (whether it involves dysfunction of the Blast valve, alterations in the endolymphatic sac or duct, or changes at the cochlear level) remains unresolved by this analysis. Additionally, the lack of a consistent correlation between the disease phenotype or onset pattern and a specific hydrops presentation suggests the involvement of other, as-yet-undiscovered non-clinical biomarkers in these processes.

Another intriguing characteristic of Ménière’s disease (MD) is its mode of initiation, which is often incomplete or does not present with the full symptomatic triad: simultaneous vertigo, fluctuating hearing loss, tinnitus, and ear fullness. Among our patients, the interval between the first and second true vertigo episodes was remarkably short, averaging just 3 months, allowing for an early diagnosis. In cases with an incomplete presentation, the time between the initial auditory symptoms and the first true vertigo episode (accompanied by fluctuating auditory symptoms) extended to nearly 2 years, with the second vertigo episode occurring approximately 6 months later. When vertigo alone was the initial manifestation (defined during history-taking as non-positional and spontaneous) the interval to a second true vertigo episode was slightly over 1 year. During these intervals, patients frequently reported various symptoms, leading to multiple emergency visits or scheduled specialist consultations. Notably, 20% of patients experienced positional symptoms or true positional vertigo. While this study did not aim to provide a detailed analysis of this symptomatic period, its exploration should be a focus of future research.

The TS1 and TD1 variables try to provide a better assessment of the initiation of the disease, and as a resume TS1 is probably a period of interest to inquire in our patients in future research as found in the correlation analysis with audio-vestibular tests. In a non-detailed assessment, our results are very similar to those from previous authors who performed a very detailed retrospective (43) and almost prospective evaluation of symptoms in a longer cohort of patients with MD (12). In general, as less symptoms are considered as initial it takes a longer period to develop the full spectrum of MD: as a mean 3 years for the mono-symptomatic initiation, 2 years for the bi-symptomatic (shorter when hearing loss appears with vertigo). However, the quality of the first symptom does not seem to determine the disease progression as the time to full-blown MD is almost equal in all cases, regardless of the initial symptom. Excluding for obvious reasons the delayed variant, we have found that for any clinical phenotype (idiopathic, autoimmune and with migraine) the auditory initiation is the most frequent which indicates the need for proper advice and follow-up of patients when seen just the first time for fluctuating hearing loss.

We have found that when analyzing the severity of hydrops, the incomplete initiation with vertigo and the complete form shows statistically significant results with EH in the vestibule and that the incomplete auditory (taking longer time) does not correlates to severity of cochlear neither vestibular EH. Just only age has a significant relation to the severity of cochlear hydrops which explains why hearing loss is always more severe for low-frequency shown previously when hearing loss of patients with MD is corrected to age-related hearing loss (44). This is well seen when analyzing the auditory tests and vestibular reflex evaluation: as well shown before hearing loss (as measured with the PTA) in the affected ear shows statistically significant result with the different grades of severity of EH in the cochlea and in the vestibule.

One limitation of this study is the small sample size in certain phenotype groups, such as Autoimmune and Delayed, which may affect the generalizability of the findings. The limited size of these groups reduces the statistical power to detect potential differences and trends, an important consideration when interpreting the results. Future studies with larger sample sizes would provide more robust insights and ensure a more accurate representation of these phenotypes. Despite this limitation, the statistical analysis in this study remains comprehensive and rigorous. Appropriate methods, including non parametric tests and correlation analyses, were employed to account for the characteristics of the data, thereby supporting the reliability of the conclusions despite challenges posed by the sample distribution.

## 5 Conclusion

We have observed that the initial presentation (in terms of clinical manifestations) has a very clear impact on the degree of hydrops and that the time between the first symptom and the second archetypal vertigo crisis of MD already provides a significant difference among patients. In the most frequent non-delayed sporadic variants this form of initiation does not shows significant differences or preferences for any of this.

Phenotype-specific differences, although subtle, influenced audiovestibular outcomes such as PTAaff and cVEMP responses, highlighting the importance of phenotypic characterization in understanding MD progression. Interestingly, the grade of EH itself had also significant direct impact on these audiovestibular measures, suggesting other underlying mechanisms may contribute to functional deficits. This study supports the use of precise temporal markers, such as TS1 and TR2, alongside phenotypic profiling, to better characterize early MD. For these reasons, we consider that time since the first symptom or since the second vertigo crisis should be selected to define the onset of the disease, specifying in future publications which of these elements has been used, as differences of 6 months to 2 years have been observed.

Future research should aim to further elucidate the pathophysiological pathways linking these variables, as well as the role of MRI imaging in detecting subtle variations in EH and its clinical implications.

## Data Availability

The original contributions presented in the study are included in the article/supplementary material, further inquiries can be directed to the corresponding author.
